# Organogenesis and tumorigenesis: Insight from the JAK/STAT pathway in the *Drosophila* eye

**DOI:** 10.1002/dvdy.22394

**Published:** 2010-08-24

**Authors:** Ying-Hsuan Wang, Min-Lang Huang

**Affiliations:** Department of Life Science and Institute of Molecular Biology, National Chung-Cheng UniversityChia-Yi, Taiwan

**Keywords:** *Drosophila*, eye development, organogenesis, JAK/STAT, tumorigenesis

## Abstract

The Janus kinase (JAK) signal transducer and activator of transcription (STAT) pathway is one of the main signaling pathways in eukaryotic cells. This pathway is used during diverse growth and developmental processes in multiple tissues to control cell proliferation, differentiation, survival, and apoptosis. In addition to its role during development, the JAK/STAT pathway has also been implicated in tumorigenesis. *Drosophila melanogaster* is a powerful genetic tool, and its eyes have been used extensively as a platform to study signaling pathways. Many reports have demonstrated that the JAK/STAT pathway plays pleiotropic roles in *Drosophila* eye development. Its functions and activation are decided by its interplay with other signal pathways and the epigenetic status. In this review, we focus on the functions and regulation of the JAK/STAT pathway during eye development and provide some insights into the study of this pathway in tumorigenesis. Developmental Dynamics 239:2522–2533, 2010. © 2010 Wiley-Liss, Inc.

## INTRODUCTION

Organogenesis is controlled by multiple processes such as cell growth, proliferation, and differentiation. These processes need to be regulated coordinately by complex networks of signal pathways, without which organs develop abnormally. The Janus kinase (JAK) signal transducer and activator of transcription (STAT) pathway is one of the main signaling pathways in eukaryotic cells. This pathway was first identified in interferon systems and responses to a wide range of cytokines and growth factors (Levy and Darnell, 2002). The JAK/STAT pathway is used to control cell proliferation, differentiation, survival, and apoptosis during diverse growth and developmental processes in multiple tissues (Nosaka et al., 1999; Williams, 2000; Tsai and Sun, 2004; Stephanou and Latchman, 2005). This pathway also plays a crucial role in tumorigenesis (Calo et al., 2003; Smirnova et al., 2007).

In general, activation of the JAK/STAT pathway entails the binding of an extracellular ligand to a transmembrane receptor, which causes the activation of the receptor-associated JAKs. These tyrosine kinases then phosphorylate themselves and their associated receptors to provide docking sites for the STAT transcription factors. After activation by the receptor-JAK complex, STATs translocate to the nucleus and regulate the expression of target genes. In mammals, wide and diverse types of extracellular ligands and transmembrane receptors, and many subtypes of both JAKs and STATs, are involved in the JAK/STAT pathway (Kisseleva et al., 2002).

*Drosophila* is a powerful genetic tool. The components of the pathway in *Drosophila* are relatively simple. It is composed of a single ligand family encoded by *unpaired* (*upd*) and *upd-like* genes (Harrison et al., 1998; Boulay et al., 2003), the receptor by *domeless* (*dome*; Brown et al., 2001), JAK by *hopscotch* (*hop*; Binari and Perrimon, 1994), and STAT by *Stat92E* (Hou et al., 1996; Yan et al., 1996). Because of the simplicity of the pathway in *Drosophila*, it provides a good platform for studying the pathway. The JAK/STAT pathway in *Drosophila* is implicated in segmentation, eye development, immune response, sex determination, germ/stem cell development, and heterochromatin stability (Hombria and Brown, 2002; Arbouzova and Zeidler, 2006; Li, 2008). In recent years, the *Drosophila* compound eye has been used extensively as a model system to study the functions of the JAK/STAT pathway during several cell events and tumorigenesis. Therefore, in this review, we focus on the study of the JAK/STAT pathway in *Drosophila* eye development and provide cues for further investigations of this pathway.

## THE JAK/STAT PATHWAY IN *DROSOPHILA*

**Fig. 1:**
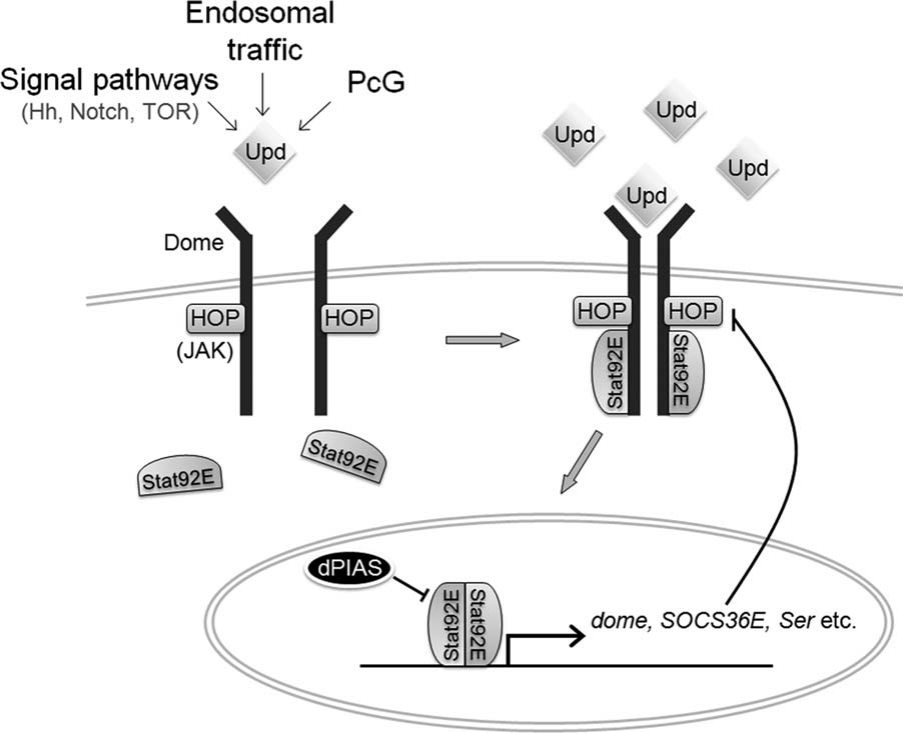
Schematic diagram of the JAK/STAT pathway in *Drosophila*. The basic components of the JAK/STAT pathway include the secreted ligand Unpaired (Upd), the transmembrane receptor Domeless (Dome), the JAK Hopscotch (Hop), and the transcription factor STAT92E. The pathway is regulated by multiple signal pathways, chromatin modification, and other regulators. Please refer to the text for details.

The JAK/STAT pathway in *Drosophila* was originally identified by means of its function in embryonic segmentation (Binari and Perrimon, 1994). Similar to what is observed in mammals, the pathway in *Drosophila* is composed of four major factors: the secreted ligand, the transmembrane receptor, the JAK kinase, and the STAT transcription factor (Hou et al., 2002). Compared with various different ligands (e.g., cytokines, interleukin, and growth factors) that trigger signaling in mammals (Subramaniam et al., 2001; Langer et al., 2004), the Upd and Upd-like proteins belong to the only ligand family that activates the JAK/STAT pathway in *Drosophila* (Boulay et al., 2003). Upd was first identified as a ligand for the JAK/STAT pathway; however, it is not homologous to any known mammalian cytokine or growth factor (Harrison et al., 1998). Originally, the Unpaired-like proteins, Upd2 and Upd3, were predicted proteins; subsequently, they were validated as ligands for the JAK/STAT pathway (Harrison et al., 1998; Hombria and Brown, 2002; Agaisse et al., 2003; Gilbert et al., 2005). In addition, the *Drosophila* genes *domeless* (*dome*), *hopscotch* (*hop*), and *Stat92E* encode proteins that are similar to the mammalian interleukin receptor, JAK, and STAT, respectively (Perrimon and Mahowald, 1986; Binari and Perrimon, 1994; Yan et al., 1996; Brown et al., 2001). Unlike that observed in mammals, no other subtypes of these components have been identified in *Drosophila*. Therefore, the JAK/STAT signaling in *Drosophila* is induced by means of the binding of Upd family ligands to Dome, which activates the JAK/STAT signaling cascade (Fig. 1).

## THE JAK/STAT PATHWAY AND *DROSOPHIAL* EYE DEVELOPMENT

### *Drosophila* Eye Development

The *Drosophila* eye has been used extensively as a model system to study the functions of signal pathways during development. The adult compound eye is derived from the eye-antennal imaginal disc of larvae (Ready et al., 1976). During the larval stage, the growth and morphogenesis of the eye disc depend on the Notch-mediated dorsal–ventral (DV) organizer, the signaling center (Dominguez and de Celis, 1998; Papayannopoulos et al., 1998). The organizer, which is established at the second instar larval stage, promotes global eye proliferation and patterning (Go et al., 1998; Kurata et al., 2000).

In the early third instar eye disc, a wavelike manner termed the morphogenetic furrow (MF) arises at the posterior margin and progresses toward the anterior margin during the third instar larval stage (Wolff and Ready, 1991). The MF initiation and progression require a complex interplay of many signal pathways, such as Decapentaplegic (Dpp), Hedgehog (Hh), Notch, and Wingless (Wg) signal pathways (Heberlein et al., 1993; Ma et al., 1993; Treisman and Rubin, 1995; Heslip et al., 1997; Greenwood and Struhl, 1999; Baonza and Freeman, 2001; Fu and Baker, 2003). In the third instar eye disc, the cells before the MF continue to proliferate, whereas the cells behind the MF begin to differentiate into mature photoreceptors (Wolff and Ready, 1991). The adult compound eye is composed of approximately 800 ommatidia. Each ommatidium is composed of eight photoreceptor cells (Ready et al., 1976). The arrangement of the photoreceptors in dorsal and ventral ommatidia display the mirror-image symmetry along the DV midline, which is termed ommatidial polarity (Chanut and Heberlein, 1995).

### Requirement for the JAK/STAT Pathway in Eye Development

Small eyes are observed in the hypomorphic *upd* loss-of-function mutant, *os^1^* (Betz et al., 2001; Chao et al., 2004). The phenotype of *os^1^* is enhanced in the genetic background of the *hop* and *Stat92E* mutants and is suppressed by overexpression of *hop^Tumorous-lethal^*, *hop^tum-l^* (Luo et al., 1999; Tsai and Sun, 2004), which encodes the constitutively active form of Hop (Hanratty and Dearolf, 1993; Luo et al., 1995). Down-regulation of JAK/STAT signaling by means of overexpression of the dominant-negative *dome*^Δ*CYT*^ (Brown et al., 2001) in the eye field reduces eye size (Tsai and Sun, 2004; Karsten et al., 2006). Consistently, the up-regulation of JAK/STAT signaling by overexpression of *upd* and *hop* causes enlargement of the eyes (Bach et al., 2003; Tsai and Sun, 2004). In addition, a growth disadvantage and defects in ommatidial polarity were found in both *hop* and *Stat92E* mutant clones (Luo et al., 1999; Zeidler et al., 1999). Thus, the JAK/STAT pathway is required for normal eye development.

### Activation of the JAK/STAT Pathway During Eye Development

**Fig. 2:**
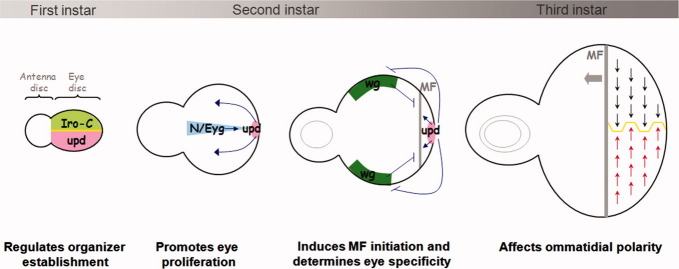
Functions of the JAK/STAT pathway in *Drosophila* eye development. The JAK/STAT pathway plays various roles during eye development. This schematic diagram shows the eye disc at different larval stages and the corresponding functions of the JAK/STAT pathway. Details are described in the text. The eye-antennal discs are drawn with anterior to the left and dorsal to the top. Light green, *Iro-C* expression; Pink, *upd* expression; yellow lines, dorsal–ventral midline; light blue, Notch/Eyg signaling; dark green, *wg* expression; gray lines, morphogenetic furrow (MF); gray arrow, MF progression; black and red arrow, ommatidial polarity.

The JAK/STAT pathway is activated in eye tissues during development (Zeidler et al., 1999; Tsai and Sun, 2004), and this activation is dependent on the presence of the ligand Upd. The expression of Upd is temporally and spatially regulated during eye development. In the first and early second instar eye discs, *upd* is expressed in the ventral eye (Reynolds-Kenneally and Mlodzik, 2005; Gutierrez-Avino et al., 2009). At the late second to early third instar stage, *upd* is expressed at the posterior boundary of the DV midline, which is termed the posterior center (PC). After MF initiation, *upd* expression at the PC is turned off (Zeidler et al., 1999; Tsai and Sun, 2004; Fig. 2).

The *10XStat92E-GFP* reporter has been used to reflect JAK/STAT activity (Bach et al., 2007), and it has been revealed that, during the first to early second instar larval stages, the JAK/STAT pathway is activated at the ventral eye disc, slightly broader than the region of Upd expression (Gutierrez-Avino et al., 2009). During the second to early third instar larval stages, the pathway is activated in the entire eye disc (Bach et al., 2007). Of interest, this activation progressively decreases, depending on the distance from the PC, where Upd is expressed. It is noteworthy that the regions of the JAK/STAT activity are consistent with the dynamic expression and the secretion of Upd. In late third instar eye discs, the JAK/STAT pathway is no longer active (Zeidler et al., 1999; Tsai and Sun, 2004; Bach et al., 2007).

Although Upd2 and Upd3 are reported as ligands for the JAK/STAT pathway (Agaisse et al., 2003; Gilbert et al., 2005; Hombria et al., 2005), whether they play a role in eye development remains unknown.

## THE FUNCTIONS OF THE JAK/STAT PATHWAY IN EYE DEVELOPMENT

### Organizer Establishment

The JAK/STAT pathway displays different functions at different stages during eye development (Fig. 2). The Notch-mediated DV organizer is established during the early second instar larval stage (Dominguez and de Celis, 1998; Papayannopoulos et al., 1998) by means of the asymmetric distribution of the dorsal-compartment selector genes of the *Iroquois complex* (*Iro-C*; McNeill et al., 1997; Cavodeassi et al., 1999). The JAK/STAT pathway is activated in the ventral eye disc at the first to early second instar larval stage (Reynolds-Kenneally and Mlodzik, 2005; Gutierrez-Avino et al., 2009). Overexpression of *upd* at the early stage results in dorsal eye overgrowth (Bach et al., 2003). Gutierrez-Avino et al. indicated that the overgrowth is associated with ectopic organizer formation (Gutierrez-Avino et al., 2009), which results from the repression on *Iro-C* distribution by the early activity of Upd. Inhibition of the JAK/STAT signaling also disturbed the *Iro-C* expression at DV, suggesting that the quantity of JAK/STAT activity is important for normal *Iro-C* distribution and organizer establishment. Furthermore, epistatic analyses have demonstrated that JAK/STAT signaling induces growth upstream from the Notch-mediated DV organizer (Gutierrez-Avino et al., 2009). Hence, the JAK/STAT pathway activated in the ventral eye disc at the early stage acts upstream from the Iro-C and Notch signaling to regulate organizer establishment.

### Proliferation

Hyperactivation of the JAK/STAT signaling causes the large-eye phenotype (Bach et al., 2003; Tsai and Sun, 2004). Of interest, when *upd* is clonally overexpressed in the eye disc, nonautonomous cell proliferation is detected only in the areas anterior to the MF (Tsai and Sun, 2004). Generating *dome^ΔCYT^* overexpression clones reduces the number of proliferating cells before the MF (Tsai and Sun, 2004). These findings suggest that JAK/STAT signaling can promote proliferation of cells anterior to the MF.

Notch/Eyegone (Notch/Eyg) signaling is activated exclusively at the DV boundary but can promote whole-eye growth at the second instar larval stage (Dominguez and de Celis, 1998; Papayannopoulos et al., 1998), suggesting the existence of a mediator that promotes the global growth. Indeed, transcription of *upd* is induced at PC by Notch/Eyg signaling (this will be discussed later). Overexpression of *upd* by *dpp-GAL4* (Staehling-Hampton et al., 1994) rescues the “eye absent” phenotype of the *eyg* mutant, and the *os^1^* mutant suppresses the large-eye phenotype caused by overexpression of *eyg* (Chao et al., 2004). Thus, Chao et al. concluded that the JAK/STAT pathway promotes cell proliferation and mediates Notch/Eyg-induced eye growth at the second instar larval stage.

### MF Initiation and Eye Specificity

Wg is homologous to mammalian Wnt protein. During *Drosophila* eye development, the Wg pathway functions in specifying head capsule development (Royet and Finkelstein, 1996) and as a negative regulator of MF progression (Ma and Moses, 1995; Baonza and Freeman, 2002). Of interest, the generation of *Stat92E* mutant clones at the larval stage leads to the formation of ectopic cuticle or ambiguous structures in adult eyes (Ekas et al., 2006). Furthermore, MF initiation is suppressed when the JAK/STAT pathway is down-regulated (Tsai et al., 2007). The *upd* overexpression clone induces ectopic MF initiation and suppresses *wg* expression. The ectopic MF initiation can be suppressed when *wg* is replenished. These observations raise the possibility that the JAK/STAT pathway positively regulates eye specificity and MF progression by means of the repression of *wg*.

Indeed, there are evidences that *Stat92E* and *hop* mutants exhibit ectopic *wg* expression in the eye discs, and endogenous *wg* expression is repressed in the *hop* overexpression clone (Ekas et al., 2006; Tsai et al., 2007). However, *wg* eye enhancers are devoid of any STAT92E binding site, suggesting that STAT92E may not directly regulate *wg* (Hou et al., 1996; Ekas et al., 2006; Tsai et al., 2007). Therefore, the JAK/STAT pathway represses the transcription of *wg* possibly by means of an indirect mechanism to initiate MF progression and to determine eye specificity.

### Ommatidial Polarity

The previous studies demonstrated that the ommatidial polarity is altered when JAK/STAT activity is misregulated (Luo et al., 1999; Zeidler et al., 1999). For instance, induction of *hop* mutant clones and *upd* overexpression clones during early eye development results in inversions of ommatidial orientation. These findings suggest that the JAK/STAT pathway is involved in the determination of polarity. However, at the third instar larval stage, when ommatidial polarity begins to be established, the JAK/STAT signaling observed by *10XStat92E-GFP* is no longer activated (Tsai and Sun, 2004; Bach et al., 2007). How does the JAK/STAT pathway control ommatidial polarity? Because the formation of the DV midline, which decides the ommatidial polarity (McNeill et al., 1997; Dominguez and de Celis, 1998), requires JAK/STAT signaling during early eye development (Flaherty et al., 2009; Gutierrez-Avino et al., 2009), the early JAK/STAT signaling may indirectly contribute to ommatidial polarity by means of regulating the establishment of the DV midline (Gutierrez-Avino et al., 2009). Alternatively, JAK/STAT signaling may regulate ommatidial polarity through a noncanonical mechanism, which is independent of the STAT92E transactivation activity (Shi et al., 2006, 2008b).

## REGULATION OF THE JAK/STAT PATHWAY BY SIGNAL PATHWAYS

The next subject is an explanation of how the JAK/STAT pathway is regulated during eye development. Signal pathways always interact with each other as a network. Some signal pathways regulating the JAK/STAT pathway have been reported, including the Hh, Notch, and target of rapamycin (TOR) pathways. Besides, the JAK/STAT pathway performs a feedback loop to regulate itself (Fig. 1).

### Hh Pathway

The *hh* gene is expressed in the ventral eye disc during the first instar stage but is expressed in the entire posterior margin from the late second to third instar stage (Cho et al., 2000). This dynamic expression resembles that of *upd*, with the exception that *upd* is expressed specifically at the PC but not in the entire posterior margin at the third instar stage, which suggests a correlation between these two pathways. The Hh pathway is activated as Hh binds to, and inhibits, Patched (Ptc), allowing the transmembrane protein Smoothened to activate the downstream transcription factor Cubitus interruptus (Ci; Ingham and McMahon, 2001). *upd* ectopic expression can be induced when *ptc* mutant clones are generated at the lateral margin or DV boundary, suggesting that the Hh signaling induces *upd* expression. However, the *ptc* mutant clones that do not reside at the lateral margin or at the DV boundary are unable to induce *upd* expression (Reifegerste et al., 1997), indicating that other factors are involved in the regulation of *upd* expression.

### Notch Pathway

The Upd expression at the PC raises the possibility that the signal at the DV midline may regulate *upd* transcription positively. Notch signaling is activated and turns on *eyg* transcription at the DV midline in the second instar eye disc (Dominguez and de Celis, 1998; Papayannopoulos et al., 1998; Agaisse et al., 2003; Jang et al., 2003; Chao et al., 2004; Dominguez et al., 2004). Previous reports demonstrated that *upd* transcription is inhibited in *Notch* mutant clones and dominant-negative Notch-expressing clones, and that the loss of *eyg* also results in the down-regulation of *upd* transcription at the PC (Chao et al., 2004; Reynolds-Kenneally and Mlodzik, 2005). This suggests that the Notch/Eyg pathway acts upstream of the JAK/STAT pathway to activate the *upd* expression at the PC.

Conversely, recent reports found that regulation of the JAK/STAT pathway upstream from the Notch pathway (Flaherty et al., 2009; Gutierrez-Avino et al., 2009). *Serrate* (*Ser*), which encodes a Notch ligand, is specifically expressed in the ventral eye disc (Papayannopoulos et al., 1998). Its transcription in the eye disc is repressed upon Upd overexpression (Flaherty et al., 2009). Additionally, the transcription of *Ser* is induced in *Stat92E* mutant clones, and it accompanies ectopic activation of Notch/Eyg signaling at the dorsal area. This evidence indicates that the down-regulation of the JAK/STAT pathway leads to derepression of *Ser* in the dorsal eye and results in ectopic Notch/Eyg activity. Furthermore, the eye reduction observed in flies with down-regulation of the JAK/STAT pathway could be restored by overexpression of Notch intracellular domain. Upd overexpression by *eyeless-GAL4* (Hauck et al., 1999) cannot promote eye overgrowth in the *eyg* mutant background (Gutierrez-Avino et al., 2009). The epistatic analyses demonstrated that the JAK/STAT pathway possibly induces growth upstream from the Notch/Eyg pathway.

Taken together, these findings suggest that the activation of JAK/STAT pathway is regulated by Notch/Eyg signaling. However, because the JAK/STAT pathway also exhibits an ability to act upstream from Notch/Eyg signaling, how the interplay between the JAK/STAT and Notch pathways is controlled during eye development needs further investigation.

### TOR Pathway

The TOR pathway is required for the growth of the *Drosophila* eye (Oldham and Hafen, 2003). The proline-rich Akt substrate of 40 kDa (PRAS40) regulates the mammalian TOR pathway (Fonseca et al., 2007; Sancak et al., 2007; Vander Haar et al., 2007). After a decrease in the expression of the *Drosophila* PRAS40 homolog, Lobe, in the eye disc, TOR signaling is down-regulated and *upd* is ectopically expressed (Wang and Huang, 2009). In *Lobe* mutants, Notch/Eyg signaling is not hyperactivated, and the expression of Ci, Hh downstream transcription factor, is not associated with the ectopic *upd* expression, suggesting that TOR signaling may regulate the JAK/STAT pathway independently of the Hh and Notch/Eyg pathways. Overexpression of the *Drosophila TOR* (*dTOR*), which is the major factor in the TOR pathway, also results in hypoactivation of the TOR pathway (Hennig and Neufeld, 2002) and ectopic *upd* expression (Wang and Huang, 2009). Furthermore, JAK/STAT activity is elevated in *dTOR* overexpression clones (unpublished data by Wang and Huang). These data indicate that the TOR pathway acts upstream of the JAK/STAT pathway by means of the repression of *upd* expression.

### JAK/STAT Pathway

In addition to other signaling pathways, the JAK/STAT pathway may regulate itself by means of the induction of the transcription of *dome*. The *dome* gene is a transcriptional target of the JAK/STAT pathway (Ghiglione et al., 2002) and is up-regulated after *upd* overexpression in the eye (Flaherty et al., 2009). As Dome is the receptor of the JAK/STAT pathway, it is reasonable that overexpression of the Dome dominant-negative form *dome^ΔCYT^* causes eye reduction. However, overexpression of full-length Dome is unable to suppress the reduced-eye phenotype of *upd* mutants. Indeed, it renders wild-type eyes smaller (Bach et al., 2003). Therefore, the JAK/STAT pathway regulates its own activity by means of regulation of *dome* transcription; however, whether Dome negatively regulates the JAK/STAT signaling pathway needs further investigation.

## OTHER MECHANISMS REGULATING THE JAK/STAT PATHWAY

In addition to the signaling network described above, many regulators of the JAK/STAT pathway, such as SOCS36E, dPIAS, and dBRWD3, have been found in the last decade. Moreover, endosomal transport and epigenetic mechanisms also govern the pathway (Fig. 1).

### SOCS36E

The *Drosophila* SOCS36E is homologous to the mammalian suppressor of cytokine signaling (SOCS; Karsten et al., 2002), which is activated by the JAK/STAT pathway and suppresses JAK/STAT through a negative-feedback loop (Kile and Alexander, 2001; Krebs and Hilton, 2001). *SOCS36E* is expressed in eye discs. It is ectopically expressed after *upd* overexpression and has been validated as a target gene of *Stat92E* (Karsten et al., 2002; Bach et al., 2007). Overexpression of *SOCS36E* exacerbates the eye-reduction phenotype of *upd* mutants, suggesting that SOCS36E may repress JAK/STAT signaling. However, overexpression of *SOCS36E* in wild-type flies does not reduce eye size (Bach et al., 2003). The *SOCS36E* mutant identified recently (Almudi et al., 2009) could be used to determine whether the JAK/STAT pathway requires SOCS36E-mediated negative regulation during eye development.

### dPIAS

The *Drosophila* protein inhibitor of activated STAT (dPIAS) is homologous to the mammalian PIAS proteins (Hari et al., 2001), which inhibit STAT signaling by promoting STAT degradation (Chung et al., 1997; Liu et al., 1998; Ungureanu et al., 2003). Overexpression of *dPIAS* decreases eye size, and down-regulation of *dPIAS* partially suppresses the small-eye phenotype of the *upd* loss-of-function mutant, *os^1^*, suggesting that dPIAS is a negative regulator of the JAK/STAT signaling pathway (Betz et al., 2001). However, complete loss of *dPIAS* does not produce the enlarged-eye phenotype that occurs after hyperactivation of the JAK/STAT pathway. Whether the ability of dPIAS to repress JAK/STAT signaling is required for eye development remains uncertain.

### Endosomal Traffic

Precise regulation of the trafficking of ligands and receptors is critical for signal pathway transduction. It was previously shown that defects in endosomal traffic affect the JAK/STAT pathway (Moberg et al., 2005; Thompson et al., 2005; Vaccari and Bilder, 2005). Endosomal sorting complex required for transport (ESCRT) protein complexes function in sorting proteins to lysosome for degradation (Bishop and Woodman, 2001; Babst et al., 2002). In the *Drosophila* eye, the loss of ESCRT components, such as Vacuolar protein sorting 25 (Vps25, a component of ESCRT-II) and Erupted (Ept, the *vps23* homolog encoding a component of ESCRT-I), causes large-eye phenotype (Moberg et al., 2005; Vaccari and Bilder, 2005). When clones of *ept* or *vps25* were generated in the eye disc, nonautonomous cell proliferation and elevated JAK/STAT activity were detected (Moberg et al., 2005; Thompson et al., 2005; Vaccari and Bilder, 2005; Gilbert et al., 2009). Blocking JAK/STAT signaling can suppress the overgrowth phenotype, suggesting that the proliferation is mediated by means of the JAK/STAT pathway (Moberg et al., 2005; Thompson et al., 2005; Gilbert et al., 2009). Because ectopic *upd* expression and Notch accumulation are detected in both *ept* and *vps25* clones, it is reasonable to assume that the elevated JAK/STAT signaling is caused by the Notch-induced *upd* expression. Furthermore, Dome accumulation was detected in *ept* mutant clones (Moberg et al., 2005; Gilbert et al., 2009). Because Upd can increase the endocytic uptake of Dome (Ghiglione et al., 2002) and the elevated JAK/STAT signaling promotes *dome* transcription (Bach et al., 2003), the excessive Dome accumulation may be the consequence of hyperactivated JAK/STAT signaling. Alternatively, the endocytic control of Dome trafficking is critical for the activation of JAK/STAT signaling (Devergne et al., 2007). Dome accumulation may result from a defect in Ept-mediated endocytic transport and may lead to JAK/STAT hyperactivation. Taken together, these data indicate that precise endosomal transport is important for regulating the activation of the JAK/STAT pathway.

### Epigenetic Mechanisms

Multiple chromatin factors are regulators of the *Drosophila* JAK/STAT pathway (Brown and Zeidler, 2008), indicating that the pathway is regulated through epigenetic mechanisms. We focus on the factors that regulate the pathway in the eye.

### dBRWD3

The *Drosophila BRWD3* (*dBRWD3*) gene encodes the bromodomain- and WD40-containing protein (D'Costa et al., 2006). A genome-wide RNA interference screen using *Drosophila* cells revealed that *dBRWD3* is a positive regulator of the JAK/STAT pathway (Muller et al., 2005). The fly deficiency line uncovering *dBRWD3* (Bach et al., 2003) and the heterozygosity of the *dBRWD3* mutant (Muller et al., 2005) suppress the eye-enlargement phenotype caused by *upd* overexpression, suggesting that *dBRWD3* is required in the JAK/STAT pathway. dBRWD3 is a chromatin-associated protein (D'Costa et al., 2006). The human BRWD3 homolog, BRWD1, is associated with the chromatin-remodeling complex (Ramos et al., 2002; Huang et al., 2003). It is possible that dBRWD3 regulates the JAK/STAT pathway by means of chromatin remodeling.

### Heterochromatin Protein 1

The *Drosophila* JAK/STAT pathway has the noncanonical function in regulating heterochromatin stability (Shi et al., 2006, 2008b). Unphosphorylated STAT92E acts to stabilize heterochromatin by binding the heterochromatin component, heterochromatin protein 1 (HP1). Activation of the JAK/STAT pathway results in STAT92E phosphorylation, which reduces STAT92E and thus HP1 in heterochromatin, causing global heterochromatin instability. The large-eye phenotype caused by *upd* overexpression is enhanced by lowering the dosage of HP1 and is suppressed by overexpression of HP1 (Shi et al., 2006), suggesting that global heterochromatin status regulates the function of the hyperactivation of JAK/STAT pathway.

### Polycomb group

The *polycomb group* (*PcG*) genes encode proteins involved in epigenetic gene silencing (Schwartz and Pirrotta, 2007). *upd* transcription and STAT92E activity are up-regulated in *PcG* mutant eye discs, which exhibit strong overgrowth (Classen et al., 2009). Interference with the JAK/STAT signaling ameliorates the overgrowth phenotype. In addition, *upd* is a direct target of PcG-mediated gene silencing in *Drosophila* eye discs (Classen et al., 2009). PcG has also been shown to repress directly the transcription of *Notch* and *eyg* (Martinez et al., 2009), which are required for the *upd* expression at the PC (Chao et al., 2004; Reynolds-Kenneally and Mlodzik, 2005). Thus, PcG regulates *upd* expression in both direct and indirect ways. These data indicate that *upd* transcription is controlled by a PcG-mediated chromatin modification.

## FUNCTIONAL SWITCHES OF THE JAK/STAT PATHWAY

The *Drosophila* JAK/STAT pathway performs diverse functions that are dependent on the developmental timing and the genetic backgrounds.

### Developmental Timing

A functional switch of the *Drosophila* JAK/STAT pathway during development was originally found in the wing disc (Mukherjee et al., 2005). STAT92E acts to promote proliferation at the early stage but represses proliferation at the late stage. An unidentified noncanonical JAK/STAT pathway, which is not activated by Upd and Hop, accounts for the anti-proliferative role. Activating the canonical JAK/STAT pathway by overexpression of Upd or Hop can also reduce proliferation at the late stage (Mukherjee et al., 2005). The data indicate that the opposite functions of the JAK/STAT pathway appear in different developmental timing.

Similarly, in the eye disc, the expression of *Ser* that encodes a ligand of Notch is suppressed by overexpression of Upd by *GMR-GAL4* (Flaherty et al., 2009) but is ectopically induced by overexpression of Upd by *eyeless-GAL4* (Gutierrez-Avino et al., 2009). The opposite effects on the *Ser* expression may be attributed to the different timing of the overexpression, in which *GMR-GAL4* drives gene expression in cells posterior to the MF from the third instar larval stage (Hay et al., 1994); however, *eyeless-GAL4* drives gene expression in the embryonic eye primordium and in undifferentiated cells of larval eye discs (Hauck et al., 1999). The data suggest that the JAK/STAT pathway is positively and negatively regulated by Notch signaling at the early and late stages, respectively (Flaherty et al., 2009; Gutierrez-Avino et al., 2009).

### Genetic Backgrounds

The function of the JAK/STAT pathway in *Drosophila* also varies according to the genetic background. Some reports have shown that the JAK/STAT pathway has specific functions only in the absence of specific cellular factors.

### *ept* Mutant

Crumbs (Crb) is an epithelial polarity factor (Tepass et al., 1990). Excess production of Crb, which is always observed in neoplastic tissues, disrupts the architecture and polarity of epithelial tissues (Klebes and Knust, 2000; Sotillos et al., 2004). The JAK/STAT pathway is required for the *crb* expression in ovarian follicle cells and the posterior spiracles (Ghiglione et al., 2002; Lovegrove et al., 2006). However, the regulation is not seen in the wild-type eye disc because the level of the Crb protein is not altered in *Stat92E* mutant clones of the eye disc (Gilbert et al., 2009). Intriguingly, the *ept* mutant cells in eye discs display up-regulation of the JAK/STAT signaling, and the accumulation of Crb protein has been detected (Moberg et al., 2005; Gilbert et al., 2009). The mRNA level of *crb* was elevated in the *ept* mutant eye disc, but this elevation was suppressed by loss of one copy of *Stat92E* (Gilbert et al., 2009). This suggests that the hyperactivated JAK/STAT pathway is responsible for the up-regulation of *crb* expression in the *ept* mutant. Because Ept functions in endocytic transport, these findings indicate that disruption of endosomal traffic allows the JAK/STAT pathway in the eye disc to adopt the ability to regulate the *crb* expression.

### *Lobe* Mutant

The small-eye phenotype of *os^1^* is not suppressed by a reduction of apoptosis (Tsai and Sun, 2004). Additionally, a reduction of apoptosis does not affect the eye enlargement caused by *upd* overexpression (Bach et al., 2003). These findings indicate that the JAK/STAT pathway does not affect apoptosis in the eye disc. However, in *Lobe* mutants, cell apoptosis and ectopic JAK/STAT activation were observed, and they acted together to yield the small-eye phenotype (Wang and Huang, 2009). A decrease in JAK/STAT activity suppresses the apoptosis and the small-eye phenotype, suggesting that this pathway plays a role in promoting apoptosis (Wang and Huang, 2009). In other words, the JAK/STAT pathway exhibits a novel function, i.e., the promotion of cell apoptosis, in the *Lobe* mutant but not in a wild-type genetic background.

## CONSERVATION OF THE JAK/STAT PATHWAY

### The JAK/STAT Pathway in Tumorigenesis

The mutant of *hop^Tum-l^*, which encodes a hyperactivated JAK that overactivates STAT92E, has leukemia-like hematopoietic tumors (Hanratty and Dearolf, 1993; Harrison et al., 1995; Luo et al., 1995; Hou et al., 1996; Yan et al., 1996; Muller et al., 2005). In the *Drosophila* eye, overexpression of Upd causes hyperactivation of the JAK/STAT pathway, which increases the number of mitotic cells and increases eye size (Bach et al., 2003; Tsai and Sun, 2004). The hyperactivated JAK/STAT pathway caused by the loss of the tumor suppressor genes, such as *vps25* (Thompson et al., 2005) and *PcG* (Sparmann and van Lohuizen, 2006; Martinez et al., 2009), is responsible for the neoplastic phenotype in eye tissue (Vaccari and Bilder, 2005; Classen et al., 2009). Thus, the hyperactivated JAK/STAT pathway exhibits a pro-growth function and leads to tumor formation in *Drosophila*.

Aberrant activation of the JAK/STAT pathway causes pro-growth in human tumors (Calo et al., 2003; Smirnova et al., 2007; Constantinescu et al., 2008). Overexpression of constitutively active STAT3 in nude mice results in the formation of tumors (Bromberg et al., 1999), and the constitutive activation of several subtypes of STATs has been reported in many human tumors, including blood and solid cancers (Calo et al., 2003; Smirnova et al., 2007). Hyperactive and constitutive forms of JAK mutations have been identified in human hematopoietic malignancies (Constantinescu et al., 2008). The data indicate that the pro-growth function of the hyperactivated JAK/STAT pathway is conserved and is a link to the human tumorigenesis.

The mechanism by which the hyperactivated JAK/STAT pathway performs the pro-growth function is not clear. In human cancers, the mammalian STAT proteins activate the genes promoting cell cycle progression, such as *cyclin D1* and *c-myc*, and the genes inhibiting apoptosis, such as *bcl-x* (Calo et al., 2003). Furthermore, it has been assumed that the hyperactivated JAK/STAT pathway acts through these effecters to promote cancer formation. In the *Drosophila* eye, overexpression of *upd* induces ectopic expression of Cyclin D (Bach et al., 2003; Tsai and Sun, 2004), suggesting that Cyclin D is a possible mediator for the pro-growth function of the JAK/STAT pathway. However, the large-eye phenotype caused by overexpression of *upd* is not suppressed by the loss of one copy of *cyclin D* or another core cell cycle regulatory protein (Mukherjee et al., 2006), indicating that cell cycle regulators may be not the major effecters. Finding the factor mediating the pro-growth function of the hyperactivated *Drosophila* JAK/STAT pathway may help us understand the mechanism of JAK/STAT-mediated tumorigenesis.

### Functional Switches of the JAK/STAT Pathway

Functional switches of the JAK/STAT pathway are also found in mammals. The mammalian JAK/STAT pathway can act differently depending on the developmental stages. For instance, the differentiation of astrocytes in the developing brain requires JAK/STAT signaling. However, ectopic induction of JAK/STAT signaling is able to trigger the downstream target of STAT3 only at the late stage, but not at the early stage (Takizawa et al., 2001), which indicates that the function of the JAK/STAT pathway is dependent on developmental timing.

Furthermore, the function of the mammalian JAK/STAT pathway also varies according to the genetic background. The mammalian STAT3 is generally considered a pro-growth factor, which promotes proliferation and prevents apoptosis (Stephanou and Latchman, 2005). In contrast, in the absence of SOCS3 in murine embryonic fibroblasts, which causes hyperactivation of STAT3 and dysregulates the STAT3 target gene expression, STAT3 becomes apoptotic (Lu et al., 2006). In glioblastoma, STAT3 can have opposite functions depending on the genetic background; STAT3 is tumor suppressive in the absence of the tumor suppressor PTEN but is oncogenic upon expression of the oncogenic form of the epidermal growth factor receptor EGFRvIII (de la Iglesia et al., 2008). Additionally, STAT1 acts to prevent proliferation and promote apoptosis (Stephanou and Latchman, 2005), but in some tumor cells, STAT1 plays a prosurvival role (Patterson et al., 2006; Timofeeva et al., 2006).

The *Drosophila* JAK/STAT pathway displays pro- and anti-proliferative functions at different times during wing development (Mukherjee et al., 2005), and this behavior resembles the general function of mammalian STAT3 and STAT1 (Bowman et al., 2000; Stephanou and Latchman, 2005). This may reflect the evolutionary event that the distinct functions of STAT92E are subsequently assigned to distinct vertebrate STAT proteins (Mukherjee et al., 2005). However, because having diverse functions in different statuses is found in both *Drosophila* and mammalian JAK/STAT pathways, the phenomena may point to a common property, in which the JAK/STAT pathway can act differently when genetic background and developmental timing are changed.

## FUNCTIONAL DECISIONS OF THE JAK/STAT PATHWAY

How does the JAK/STAT pathway play distinct roles at different developmental times and genetic backgrounds? In the case of astrocyte differentiation, STAT3 directly binds and activates the gene *gfap*, which encodes the astrocyte marker glial fibrillary acidic protein (GFAP; Bonni et al., 1997; Song and Ghosh, 2004). The accessibility of STAT3 in the *gfap* promoter is regulated by the developmental stage-dependent DNA methylation (Takizawa et al., 2001), fibroblast growth factor 2-dependent histone methylation (Song and Ghosh, 2004), and retinoic acid-dependent histone H3 acetylation (Asano et al., 2009). The evidence indicates that, during development, chromatin structures controlled by signaling pathways determine the function of the JAK/STAT pathway.

The aforementioned findings show that Notch, Hh, and TOR signal pathways can regulate the JAK/STAT pathway; however, the detail of the mechanism is still unclear. Mammalian Notch intracellular domain has functional interaction with histone acetylases and assists in chromatin remodeling (Kurooka and Honjo, 2000). It is possible that these signal pathways regulate the JAK/STAT pathway by means of epigenetic mechanisms. Additionally, it is known that PcG can directly suppress *upd* and *Notch* transcription, indicating that an epigenetic mechanism can regulate JAK/STAT activation directly or through other signal pathways.

On the other hand, the JAK/STAT pathway can act conversely to regulate other signaling pathways, such as Notch and Wg (Ekas et al., 2006; Tsai et al., 2007; Flaherty et al., 2009; Gutierrez-Avino et al., 2009), and can affect global epigenetic status per se. In *Drosophila*, constitutive JAK/STAT signaling disrupts heterochromatin structures and affects the genes that are not the direct target of STAT (Shi et al., 2006). Mammalian STAT proteins recruit chromatin-modifying enzymes such as histone acetyltransferases (Schindler et al., 2007). Activation of the mammalian JAK/STAT pathway can trigger chromatin remodeling (Christova et al., 2007; Shi et al., 2008a).

**Fig. 3:**
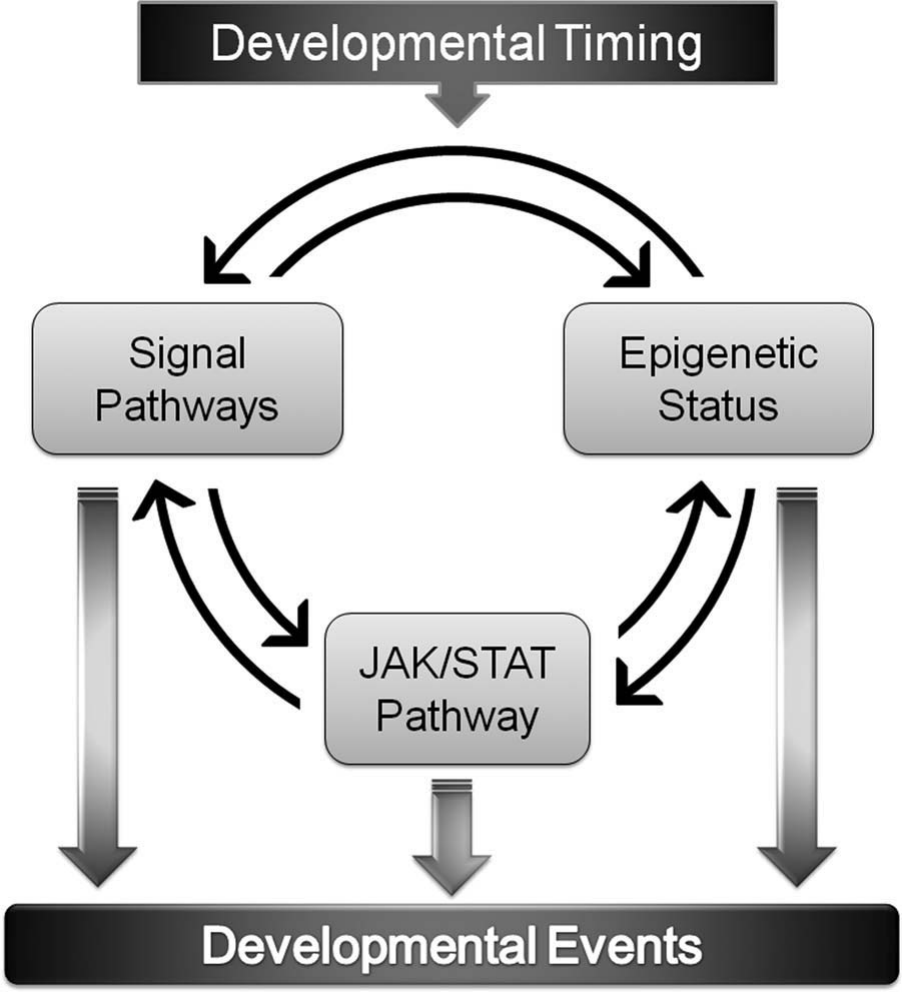
Model of the interplay among the JAK/STAT pathway, other signal pathways, and epigenetic status. During development, the JAK/STAT pathway, other signal pathways (such as Notch pathway), and epigenetic status cooperate to influence each other. The interplay determines the activation and functions of the JAK/STAT pathway and, ultimately, developmental events.

These findings lead to a model in which the JAK/STAT pathway, epigenetic status, and other signal pathways are regulated by each other in an interdependent network (Fig. 3). During normal development, the JAK/STAT pathway is activated by signaling pathways and epigenetic mechanisms, both of which cooperatively determine the function of the JAK/STAT pathway. After being activated, the JAK/STAT signaling conversely alters the other signaling pathways and chromatin structures, which thus adjust the functions of the JAK/STAT pathway at later stages. Finally, the negative factors such as SOSC36E act together with signaling pathways and chromatin status, which suppress *upd* transcription at this stage, to turn off the JAK/STAT signaling. Upon loss of *ept* or *Lobe*, hyperactivated JAK/STAT signaling and dysregulated Notch or TOR pathways disrupt the balance of the network, resulting in aberrant functions of the JAK/STAT pathway. Therefore, the interplay among the JAK/STAT pathway, epigenetic status, and other signaling pathways controls the strength, duration, and output of the JAK/STAT signaling.

## FUTURE PERSPECTIVES

Several questions remain unanswered regarding the functions and regulations of the JAK/STAT pathway. First, it remains to be determined whether the JAK/STAT pathway affects normal eye development by controlling chromatin structures. Second, do developmental timing and signal pathways control the transcription of *upd* through epigenetic mechanisms? The epigenetic status of the *upd* locus in different developmental stages and in the mutation of genes, such as *Hh*, *Notch*, *ept*, *vps25*, and *Lobe*, which regulate *upd* transcription, should be investigated. It is also interesting to study whether the other signaling pathways regulate *upd* transcription through PcG.

Next, if the function of the hyperactivated JAK/STAT pathway differs from that of the endogenous one, the data of hyperactivated JAK/STAT signaling should be carefully interpreted. For example, whether the factors modifying the phenotype of hyperactivated JAK/STAT pathway also participate in the endogenous pathway should be confirmed. Whether the pro-growth function of the JAK/STAT pathway is mediated by alteration of epigenetic status and other signal pathways awaits further investigation.

Moreover, to investigate whether the diverse functions of the *Drosophila* JAK/STAT pathway in different developmental stages and in the aforementioned conditions are attributed to epigenetic alteration and dysregulation of global gene expressions, the further examinations, such as genome-wide analyses of epigenetic status, transcriptome, and the expression of the STAT92E target genes, are required.

The function and regulation of the JAK/STAT pathway observed in the *Drosophila* eye may be evolutionarily conserved in mammals. For instance, mammalian interleukins, like the Upd signal through the JAK/STAT pathway, are also regulated by the Notch and the TOR pathways (Amsen et al., 2004; Weichhart et al., 2008). In addition, SOCS and PIAS are well-known modulators of the mammalian JAK/STAT pathway (Shuai, 2006; Croker et al., 2008). The signal pathways and epigenetic mechanisms regulating the *Drosophila* JAK/STAT pathway have been implicated in tumorigenesis (Wicking and McGlinn, 2001; Faivre et al., 2006; Leong and Karsan, 2006; Sparmann and van Lohuizen, 2006). Furthermore, it has been proposed that hyperactivation of the JAK/STAT pathway in cancers may alter global chromatin conformation, leading to dysregulation of nontarget genes (Constantinescu et al., 2008). It is possible that the interplay among the JAK/STAT pathway, other signal pathways, and epigenetic status (Fig. 3) contributes to the activation and functions of the JAK/STAT pathway in human cancers. Thus, examination of the hypothesis will provide valuable insights into the biology of cancer.

Human tumors have genetic heterogeneity (Heppner, 1984). The complex interactions between tumor cells and the adjacent cells are important for tumorigenesis (Kinzler and Vogelstein, 1996; Hanahan and Weinberg, 2000). The *Drosophila* eye is a well-established platform for clonal analyses (Xu and Rubin, 1993; Lee and Luo, 1999), which are used to study the interactions of the neighboring cells of different genetic backgrounds. Therefore, the investigations using the *Drosophila* eye may provide insights into the role of the JAK/STAT pathway in organogenesis and tumorigenesis.
